# Reviewed article: Tauil MC, Castro APB, Andrade SSCA, Freitas FTM, Moura NFO. Non-COVID-19 respiratory tract infection mortality among children under 5 years old: a time series, Brazil, 2002-2022. Epidemiol Serv Saude. 2026:35;e20250575.

**DOI:** 10.1590/S2237-96222026v35e20250575.a

**Published:** 2026-04-10

**Authors:** Ana Elisa Madalena Rinaldi

**Affiliations:** 1 Universidade Federal de Uberlândia, Uberlândia, MG, Brazil Universidade Federal de Uberlândia Uberlândia MG Brazil

## Peer review A

## Questionnaire

Does the manuscript contain new and significant information to justify publication? No

Does the Abstract (Summary) clearly and accurately describe the content of the article? Yes

Is the problem significant and concisely stated? Yes

Are the methods described comprehensively? Yes

Are the interpretations and conclusions justified by the results? Yes

Is adequate reference made to other work in the field? Yes

## Manuscript Structure

Length of article is: Adequate

Number of tables is: Adequate

Number of figures is: Adequate

Please state any conflict(s) of interest that you have in relation to the review of this paper. None

Interest: Good

Quality: Good

Originality: Good

Overall: Good

Recommendation: Minor Revision

## Comments

O presente manuscrito tem como objetivo principal descrever e analisar a tendência temporal dos óbitos por infecções respiratórias associadas à bronquiolite e vírus sincicial respiratório em crianças brasileiras menores de cinco anos no período de 2002 a 2022. É um estudo ecológico, cujos dados foram provenientes do Sistema de Informação de Mortalidade (SIM), obtidos na Plataforma de Ciência de dados aplicação à saúde da Fundação Oswaldo Cruz. Os autores também utilizaram os dados dos números de nascidos vivos disponíveis no site do DataSUS. O manuscrito está estruturado em introdução, métodos, resultados e discussão, com um parágrafo final de conclusões finais. Os resultados se alinham aos métodos e objetivos propostos. As principais sugestões que julgo poderem contribuir para a melhoria do texto (forma de organização dos dados na introdução, métodos e resultados) estão descritas segundo as seções do manuscrito.

Introdução:

Na introdução, os/as autores/as fazem uma descrição da mortalidade por doenças respiratória no Brasil, no mundo e na América Latina e apresentam uma breve justificativa do estudo. Minhas sugestões na introdução são duas questões em especial:

-Reorganizar os parágrafos, de forma a contemplar inicialmente o panorama da mortalidade por doenças respiratórias na primeira infância no mundo (global), posteriormente na América Latina e no Brasil. Não fica muito clara a razão de citar especificamente dois estudos de vigilância populacional realizado nos Estados Unidos e no Equador (uma possível explicação seria a adoção da vacinação contra Streptococcus pneumoniae, porém precisa deixar mais contextualizado com o restante dos dados da introdução). Contextualizar mais diretamente os dados de vacinação e da tendência, se esse for o foco escolhido pelos/as autores/as.

-Apresentar uma justificativa mais consistente para o desenvolvimento do estudo. A ideia seria entender o contexto nacional anterior à nova vacinação contra o vírus sincicial em gestantes? 

Métodos:

O desenho do estudo, fonte dos dados e as variáveis utilizadas estão descritas de forma clara.

Algumas sugestões:

-Subitem “Contexto”: o período analisado no artigo é de 2002 a 2022 e neste tópico, há a descrição de características demográficas do país em 2023. Desta forma, minha sugestão seria fazer uma descrição com dados que se refere ao período analisado (2002 a 2022).

-Subitem “Variáveis”: há a descrição de algumas variáveis que, em função do percentual elevado de dados ignorados, não foram descritas em resultados (faixa etária e escolaridade materna, quantidade de filhos nascidos vivos, tipo de parto, tipo de gravidez, semanas de gestação). Como não foram analisados, acho que não precisam ser descritos no manuscrito. Minha sugestão seria retirar dos métodos e dos resultados (linhas 45-49, página 4). Citar nas limitações do estudo (variáveis que foram inicialmente coletadas dos bancos de dados, mas tinham um elevado percentual de valores ignorados). Neste tópico, descrever de forma geral, o percentual de valores ignorados nas variáveis.

-Subitem “métodos estatísticos”: incluir a descrição de que os dados foram descritos segundo Brasil e regiões geográficas (conforme consta na Tabela 1).

Resultados:

Os resultados estão descritos de forma clara no texto e apresentados de forma clara nas teblas e figuras. No primeiro parágrafo a sugestão seria tecer comentários sobre semelhanças e diferenças regionais para aas variáveis apresentadas. A variável “peso ao nascer” tem um elevado percentual de valores ignorados (mais de 50%), talvez não seja tão interessante fazer a apresentados dos resultados para esta variável, assim como foi adotado para aquelas que tinham mais de 50% dos valores ignorados.

No terceiro parágrafo (linhas 6-15, página 5) tem dados que não estão descritos em tabelas ou figuras. Minha sugestão seria para os autores concentrarem as informações que estão em tabelas/figuras e posteriormente sinalizar quais não estão e deixar sinalizado entre parênteses (dados não apresentados em tabelas e figuras).

Figura 2: a sugestão seria colocar uma letra para cada gráfico - Figura 2A-observações; Figura 2B-tendência; Figura 2C-Sazonalidade; Figura 2D-Aleatoriedade. Desta forma, a apresentação dos resultados no texto fica mais direta.

No quarto parágrafo (linhas 39-43, página 5): há o destaque para o ano de 2022. A comparação foi em comparação à 2002?

Discussão:

Segundo parágrafo (linhas 5-10, página 6) - Nas limitações do estudo, poderia ser acrescentado uma sensibilização dos profissionais da saúde para o preenchimento dos dados, em função do percentual elevado de dados ignorados.

Acho que seria importante fazer um parágrafo destacando as políticas de saúde que também possam ter contribuído com a redução da mortalidade por doenças respiratórias, assim como destacar alterações no calendário vacinal. Incluir a citação da nova vacina (vacina contra o vírus sincicial para gestantes) neste parágrafo.

Parágrafo de conclusão: talvez a citação da nova vacina poderia ser citada neste parágrafo sugerido acima.

